# A dual-feedback loop model of the mammalian circadian clock for multi-input control of circadian phase

**DOI:** 10.1371/journal.pcbi.1008459

**Published:** 2020-11-23

**Authors:** Lindsey S. Brown, Francis J. Doyle

**Affiliations:** 1 Harvard John A. Paulson School of Engineering and Applied Sciences, Harvard University, Cambridge, Massachusetts, United States of America; 2 Division of Sleep Medicine, Harvard Medical School, Boston, Massachusetts, United States of America; University of Pittsburgh, UNITED STATES

## Abstract

The molecular circadian clock is driven by interlocked transcriptional-translational feedback loops, producing oscillations in the expressions of genes and proteins to coordinate the timing of biological processes throughout the body. Modeling this system gives insight into the underlying processes driving oscillations in an activator-repressor architecture and allows us to make predictions about how to manipulate these oscillations. The knockdown or upregulation of different cellular components using small molecules can disrupt these rhythms, causing a phase shift, and we aim to determine the dosing of such molecules with a model-based control strategy. Mathematical models allow us to predict the phase response of the circadian clock to these interventions and time them appropriately but only if the model has enough physiological detail to describe these responses while maintaining enough simplicity for online optimization. We build a control-relevant, physiologically-based model of the two main feedback loops of the mammalian molecular clock, which provides sufficient detail to consider multi-input control. Our model captures experimentally observed peak to trough ratios, relative abundances, and phase differences in the model species, and we independently validate this model by showing that the *in silico* model reproduces much of the behavior that is observed *in vitro* under genetic knockout conditions. Because our model produces valid phase responses, it can be used in a model predictive control algorithm to determine inputs to shift phase. Our model allows us to consider multi-input control through small molecules that act on both feedback loops, and we find that changes to the parameters of the negative feedback loop are much stronger inputs for shifting phase. The strongest inputs predicted by this model provide targets for new experimental small molecules and suggest that the function of the positive feedback loop is to stabilize the oscillations while linking the circadian system to other clock-controlled processes.

## Author summary

The circadian clock helps to regulate many biological functions, including the sleep-wake cycle, metabolism, the cardiovascular system, and the immune response, so we can promote better health by aligning the internal body clock to the phase of the external environment. At the cellular level, the circadian clock is driven by the interactions of a core set of genes and proteins, presenting inputs to manipulate the clock. Mathematical models allow us to predict which inputs can best be used to shift the internal clock to align with the environment. In this paper, we develop a model, consisting of a system of differential equations, to describe the molecular level components which drive circadian rhythms and test the model to show it captures experimental observations. We then use this model to determine what parametric changes have the strongest resetting effect on the clock, suggesting which mechanisms the development of new experimental small molecules should target to most efficiently shift circadian phase. We demonstrate the efficacy of this approach in a model predictive control simulation. Although we build a model specific to the circadian clock, the techniques we describe for modeling and control of the circadian oscillator can be applied to many oscillating systems.

## Introduction

**Fig 1:**
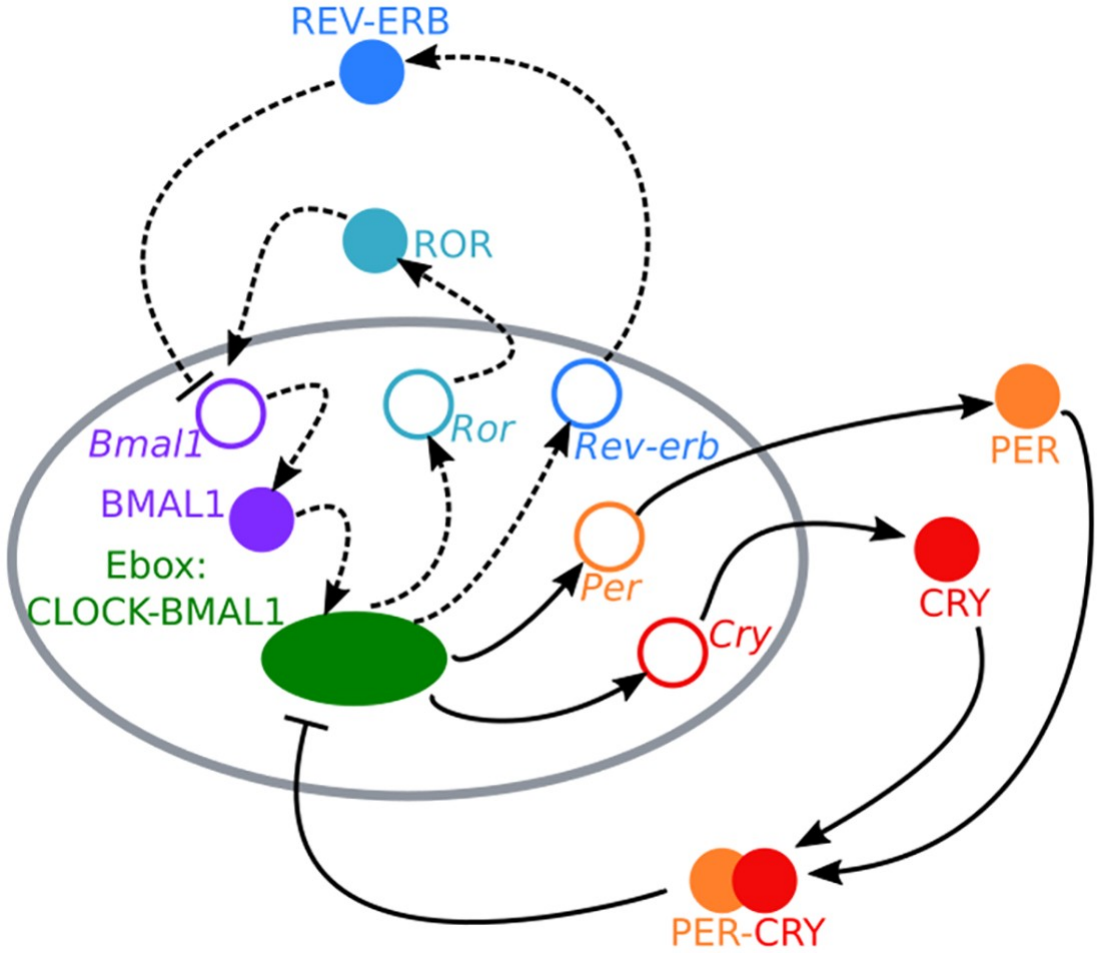
Molecular architecture of the mammalian circadian clock. Two transcription-translation feedback loops drive the oscillations of the mammalian circadian clock at the cellular level. We show the architecture for the core clock species of a single cell, where the grey oval represents the nucleus of the cell. The negative loop (warm colors, solid arrows) contains the mRNAs *Period* and *Cryptochrome* and their associated proteins. The positive loop (cool colors, dashed arrows) contains *Rev-erb*, *Ror*, and *Bmal* mRNAs and the associated proteins. Hollow circles represent mRNAs and filled circles represent proteins of the different clock species. Arrowheads represent increased production, while flat arrows represent inhibited production. The dashed lines in the positive feedback loop represent interactions that were not included in the Hirota model [1] but that were added to our extended dual loop model to provide additional control targets acting on the core circadian clock architecture.

Oscillations are a feature of many biological systems at a range of different timescales, including the cardiac cycle on the order of a second to the estrous cycle on the order of a month. For this reason, we want to identify mechanisms through which we can act on these oscillators in order to control the timing of these physiological processes. The circadian clock is an endogenous oscillator with a period of about a day, which coordinates the timing of many biological functions with the environment at the organismal and tissue levels. These higher level rhythms are driven by the molecular level clock in each cell, where two interlocked transcription-translation feedback loops form the core architecture for the mammalian circadian clock (Fig 1). The connections between these two feedback loops are exemplary of an activator-repressor system, one of the basic genetic architectures shown to produce oscillations, so can provide insight into approaches for controlling other systems with similar architectures. In particular, we aim to control the phase of the oscillator through a model-based control approach to determine the dosing of different drugs to this system, as has been shown to be successful in other medical applications such as diabetes. Such an approach requires a suitable model that captures the effects of the inputs on the controlled variables and is conducive to online optimization. Thus, by modeling these loops, we are able to analyze how each loop contributes to the resulting features of the oscillator, and as a result of the sensitivity of these features to the parameters governing the different loops conclude which processes provide the most effective targets for controlling these oscillations.

In order to build a control-relevant model of the circadian system, we focus our model on the core components of the circadian clock. In the negative feedback loop, the repressor, the mRNAs *Per* and *Cry* are transcribed in the nucleus by an Ebox bound to CLOCK:BMAL1. These genes are translated into the proteins PER and CRY in the cytoplasm where they dimerize before reentering the nucleus. The PER:CRY dimer then binds to CLOCK:BMAL1 where it represses Ebox controlled transcription. In the positive feedback loop, the activator, *Ror* and *Rev-erbα* are also regulated by the Ebox. The proteins ROR and REV-ERB*α* competitively bind to the RORE promoter site, controlling the rate of *Bmal1* transcription, which slows when REV-ERB*α* is bound and accelerates when ROR is bound [2]. This interlocked architecture of the two feedback loops results in oscillations in the expression level of the core clock genes and proteins, which then influence other genes and proteins in other biological systems to appropriately time daily functions, including the sleep wake cycle, alertness, metabolism, and the immune response. The centrality of this underlying molecular oscillator to daily function makes it critical to analyze the effects of alterations and different inputs to the clock in order to promote better health and appropriately time interventions [3].

While many mathematical models use a limit cycle oscillator to describe the circadian clock, the order of the systems of differential equations varies from basic oscillator models with only 2 to 3 states [4, 5] to models with over 70 states [6]. Such models are valuable because they allow us to make predictions about the behavior of the circadian clock in response to molecular level inputs. For example, the Hirota model [1] was used to help elucidate the action of KL001, demonstrating that it acts by decreasing the degradation rate of nuclear cryptochrome.

We are particularly interested in using these models to understand where and how to act on the molecular circadian clock to shift circadian phase, so we want a model that provides the necessary scope of control-relevant targets to identify the key processes that inputs to shift phase should target at the molecular level. This would potentially allow us to align the clock with the environment faster than light based interventions [7]. The dosing of these molecules can be formulated as a control problem, and recent work has explored the small molecule control of the circadian clock using KL001 as input using the Hirota model [1]. This model is limited in that it only describes the negative feedback loop, so we can only model small molecules, such as KL001 and longdaysin, that act on the negative arm of the clock [1, 8]. However, there are many other small molecules that have been discovered, including KK-S6, FBXW7-*α* modulators, and GSK-3*β* inhibitors, which target the species of the positive arm, which cannot be captured by this model (Table 1). Furthermore, increasing the complexity of the feedback loop structure results in models that are more flexible for achieving desired evolutionary features, including robustness of the oscillations and temperature compensation of the period length [9]. Similarly, additional loops have been implicated for helping to preserve the stoichiometric relationships needed for robust oscillations [10]. Thus, both the underlying biological behavior that our model should capture and the desire to act on the clock through multiple known targets motivate a model containing both the positive and negative feedback loops.

**Table 1:** Action of known small molecules on the molecular clock. Several small molecules are known to impact the clock by acting on core clock model species. These actions may be represented by changing a corresponding parameter of the model.

Small Molecule	Action	Model Parameter(s)
KL001 [1]	stabilizes nuclear CRY	*v*_*d*,*C*1*N*_, *v*_*d*,*C*2*N*_
Longdaysin [8]	inhibits PER degradation	*v*_*d*,*P*_
KK-S6 [11]	reinforces REV-ERB*α*	*K*_*txn*,*REV*_
FBXW7-*α* [12]	degrades REV-ERB*α*	*v*_*d*,*REV*_
GSK3-*β* inhibitors [13]	degrades CRY2, stabilizes REV-ERB*α*	*v*_*d*,*C*2_, *v*_*d*,*REV*_

For this reason, we developed a more detailed model of the mammalian molecular circadian oscillator, expanding the eight state Hirota model [1] of the negative feedback loop to a 14 state model with both feedback loops so that the model is just complex enough to capture the action of these additional inputs. Thus, our model focuses on producing the essential behavior necessary for suitable control action while retaining enough simplicity so that we can identify the key processes which are needed to generate circadian oscillations and increasing the tractability of determining the most effective inputs to shift the clock. Once these key processes have been identified, more detailed models of these specific mechanisms could be employed for the development of small molecule inputs to the clock. We outline the advantages of our model over other models of similar size in the discussion. One advantage of this model is that validation showed that this expanded model captures much of the behavior of the core circadian clock. The new model allowed us to explore ways to enhance control of the circadian oscillator through a multi-input approach by providing additional known targets for control input that act on both feedback loops of the clock, which are omitted in some previous models or do not show the correct knockout behavior in other models. By comparing the sensitivity of the model period to the different model parameters, we determined which parameters would be most effective in controlling circadian phase. We found that sensitivities to the parameters governing the negative feedback loop were much higher, suggesting that these would be stronger targets for control and implicating the role of the positive feedback loop for robustness and in the timing of other biological functions.

## Materials and methods

### Equation derivation

We developed a model to describe the transcription, translation, and degradation of the core clock mRNAs and proteins, which constitute the core positive and negative feedback loops of the clock. We used a biologically based model, which allows for the parameters of the model to be interpreted in terms of different processes so that the action of different small molecules can be reflected by changing a model parameter. For example, the small molecule KL001 decreases the degradation rate of nuclear cryptochrome, so by modifying the parameter corresponding to this rate, we can simulate the action of this small molecule with our model.

The Hirota model [1] successfully captured the action of this small molecule, but the model was limited only to the eight species of the negative feedback loop, preventing the analysis of the known small molecules that act on the positive feedback loop for control. In order to expand this model from the original eight states to our fourteen state model of both feedback loops, we analogously assumed that the mRNA and protein species decay according to Michaelis-Menten kinetics, since experimental evidence has found that enzymes contribute to the decay of these species, and that all other reactions follow mass action kinetics. In this way, each parameter of our model can be related to a specific biological process, allowing the model to be physiologically interpretable, which is beneficial in identifying which pathways new drugs should target. By explicitly modeling the gene, protein, and heterodimer of cryptochrome, we have created a series of states which results in the time delay known to be required for oscillations but without explicitly specifying a time delay as in other models [14]. While models with explicit time delays may require fewer states and parameters, these models lose physiological interpretability and are more challenging for a multivariable model-based control framework.

The key difference between our model and the previous Hirota model [1] is the incorporation of the species in the positive feedback loop, which requires modification to several of the equations. First, unlike in the previous model, we found that we do not need to introduce a Hill term to the degradation because the addition of the positive feedback loop to our model provided sufficient nonlinearity to drive oscillations [15]. Second, since the Hirota model [1] did not explicitly model BMAL1, it was assumed that there was always sufficient concentrations of CLOCK:BMAL1 to bind the Ebox. Our model incorporated *Bmal1* and BMAL1 so this is no longer a valid assumption, and we derived an expression for the transcription of the Ebox controlled genes (*Per*, *Cry1*, *Cry2*, *Rev-erbα*, and *Ror*). Assuming the biochemical reactions are at equilibrium, we found the transcription rate to be

$$\begin{array}{c} r=\frac{v_{r}}{K_{r}+\frac{K_{b}}{\left[B\right]}+\left[C1N\right]+\left[C2N\right]}, \end{array}$$ (1)

where *v*_*r*_, *K*_*r*_, and *K*_*b*_ are constant model parameters and [*B*], [*C*1*N*], and [*C*2*N*] are the concentration of BMAL1 and the heterodimers of CRY1 and CRY2 with PER in the nucleus. The form of this expression showed that BMAL1 is necessary for the production of the Ebox regulated mRNAs to create oscillations.

In order to describe the transcription of *Bmal1*, we had to account for the competitive binding of REV-ERB*α* and ROR to the RORE binding site of the promoter. When REV-ERB*α* is bound to this site, the rate of transcription of *Bmal1* is reduced by 57–80% of the unbound rate, but when ROR binds to this site, the transcription rate is significantly increased [2]. Again assuming equilibrium, we derived the rate of transcription of *Bmal1* to be

$$\begin{array}{c} r_{b}=\frac{v_{1}\left[ROR\right]+v_{2}}{1+K_{1}\left[REV\right]+K_{2}\left[ROR\right]}, \end{array}$$ (2)

where *v*_1_, *v*_2_, *K*_1_, and *K*_2_ are constants, and [*ROR*] and [*REV*] are the concentrations of ROR and REV-ERB*α*.

Using these expressions for the transcription rates and the assumptions of Michaelis-Menten kinetics and mass action kinetics, we formed a fourteen state ordinary differential equation model to describe the evolution of the concentration of our model species, given by the following system of equations:

$$\begin{array}{c} \frac{d[p]}{dt}=\frac{v_{txn,p}}{K_{txn,p}+\frac{K_{b}}{\left[B\right]}+\left[C1N\right]+\left[C2N\right]}-\frac{v_{deg,p}\left[p\right]}{K_{deg,p}+\left[p\right]} \end{array}$$ (3)

$$\begin{array}{c} \frac{d[c1]}{dt}=\frac{v_{txn,c1}}{K_{txn,c}+\frac{K_{b}}{\left[B\right]}+\left[C1N\right]+\left[C2N\right]}-\frac{v_{deg,c1}\left[c1\right]}{K_{deg,c}+\left[c1\right]} \end{array}$$ (4)

$$\begin{array}{c} \frac{d[c2]}{dt}=\frac{v_{txn,c2}}{K_{txn,c}+\frac{K_{b}}{\left[B\right]}+\left[C1N\right]+\left[C2N\right]}-\frac{v_{deg,c2}\left[c2\right]}{K_{deg,c}+\left[c2\right]} \end{array}$$ (5)

$$\begin{array}{c} \frac{d[ror]}{dt}=\frac{v_{txn,ror}}{K_{txn,ror}+\frac{K_{b}}{\left[B\right]}+\left[C1N\right]+\left[C2N\right]}-\frac{v_{deg,ror}\left[ror\right]}{K_{deg,ror}+\left[ror\right]} \end{array}$$ (6)

$$\begin{array}{c} \frac{d[rev]}{dt}=\frac{v_{txn,rev}}{K_{txn,rev}+\frac{K_{b}}{\left[B\right]}+\left[C1N\right]+\left[C2N\right]}-\frac{v_{deg,rev}\left[rev\right]}{K_{deg,rev}+\left[rev\right]} \end{array}$$ (7)

$$\begin{array}{c} \frac{d[P]}{dt}=K_{tln,p}\left[p\right]-\frac{v_{deg,P}\left[P\right]}{K_{deg,P}+\left[P\right]}-v_{a,CP}\left[P\right]\left[C1\right]+v_{d,CP}\left[C1N\right]\\ -v_{a,CP}\left[P\right]\left[C2\right]+v_{d,CP}\left[C2N\right] \end{array}$$ (8)

$$\begin{array}{c} \frac{d[C1]}{dt}=K_{tln,c1}\left[c1\right]-\frac{v_{deg,C1}\left[C1\right]}{K_{deg,C}+\left[C1\right]}-v_{a,CP}\left[P\right]\left[C1\right]+v_{d,CP}\left[C1N\right] \end{array}$$ (9)

$$\begin{array}{c} \frac{d[C2]}{dt}=K_{tln,c2}\left[c2\right]-\frac{v_{deg,C2}\left[C2\right]}{K_{deg,C}+\left[C2\right]}-v_{a,CP}\left[P\right]\left[C2\right]+v_{d,CP}\left[C2N\right] \end{array}$$ (10)

$$\begin{array}{c} \frac{d[ROR]}{dt}=K_{tln,ror}\left[ror\right]-\frac{v_{deg,ROR}\left[ROR\right]}{K_{deg,ROR}+\left[ROR\right]} \end{array}$$ (11)

$$\begin{array}{c} \frac{d[REV]}{dt}=K_{tln,rev}\left[rev\right]-\frac{v_{deg,REV}\left[REV\right]}{K_{deg,REV}+\left[REV\right]} \end{array}$$ (12)

$$\begin{array}{c} \frac{d[b]}{dt}=\frac{v_{txn,ROR}\left[ROR\right]+v_{txn,REV}}{1+K_{txn,REV}\left[REV\right]+K_{txn,ROR}\left[ROR\right]}-\frac{v_{deg,b}\left[b\right]}{K_{deg,b}+\left[b\right]} \end{array}$$ (13)

$$\begin{array}{c} \frac{d[B]}{dt}=K_{tln,b}\left[b\right]-\frac{v_{deg,B}\left[B\right]}{K_{deg,B}+\left[B\right]} \end{array}$$ (14)

$$\begin{array}{c} \frac{d[C1N]}{dt}=-\frac{v_{deg,C1N}\left[C1N\right]}{K_{deg,CP}+\left[C1N\right]+\left[C2N\right]}+v_{a,CP}\left[P\right]\left[C1\right]-v_{d,CP}\left[C1N\right] \end{array}$$ (15)

$$\begin{array}{c} \frac{d[C2N]}{dt}=-\frac{v_{deg,C2N}\left[C2N\right]}{K_{deg,CP}+\left[C1N\right]+\left[C2N\right]}+v_{a,CP}\left[P\right]\left[C2\right]-v_{d,CP}\left[C2N\right] \end{array}$$ (16)

The description of the symbols for the model species is given in Table 2. A version of the model in python, parameterized with the optimal parameter set determined as described in the next section, is available on github at https://github.com/lindseysbrown/brown_circadian_dual-feedback_loop.

**Table 2:** Model species. Definition of the symbols for the species of our model.

Symbol	Model Species
*p*	*Per* mRNA
*c*1	*Cry1* mRNA
*c*2	*Cry2* mRNA
*ror*	*Ror* mRNA
*rev*	*Rev-erbα* mRNA
*P*	PER protein
*C*1	CRY1 protein
*C*2	CRY2 protein
*ROR*	ROR protein
*REV*	REV-ERB*α* protein
*b*	*Bmal1* mRNA
*B*	BMAL1 protein
*C*1*N*	PER:CRY1 heterodimer in the nucleus
*C*2*N*	PER:CRY2 heterodimer in the nucleus

### An evolutionary algorithm for parameter fitting

For the model to be biologically relevant, we wanted the patterns of gene and protein expression levels to reflect experimental observations, by accurately reproducing peak to trough ratios, relative abundances, and phase differences of the different model species. We also required that the model preserve the correct behaviors in the *Cry1* and *Cry2* knockout cases that were produced by the Hirota model [1] since this is a desired response to a well studied control input. We used experimentally observed values, noted in Table 3, as the desired values for these features. With the exception of the *Cry1* and *Cry2* knockout cases, we did not fit our model to experimental results on the knockout of various model species, and instead, used this data to validate our model.

**Table 3:** Desired model features. The desired values of the model features with the weights used in the cost function compared with the values of these features produced by the model fit with the lowest cost parameter set.

**Peak to Trough Ratio**	**Weight**	**Desired**	**Model Feature**
*Per*	5	>20	51.19
*Cry1*	3	2.1	2.64
*Cry2*	3	2.2	2.48
*Ror*	3	4.1	3.62
*Rev-erbα*	5	>10	18.90
*Bmal1*	5	>10	2.77
PER	5	>20	487928
CRY1	3	3.7	2.66
CRY2	3	1.8	2.43
ROR	5	<5	4.12
REV-ERB*α*	5	<5	19.41
BMAL1	3	2.9	2.97
**Relative Abundance**	**Weight**	**Desired**	**Model Feature**
PER:PER+CRY1+CRY2	10	.11	.28
CRY1:PER+CRY1+CRY2	10	.56	.36
CRY2:PER+CRY1+CRY2	10	.34	.36
BMAL1:CRY1	3	.19	.17
ROR:REV	3	1.02	1.05
*Ror*:*Rev-erbα*	3	.78	.75
*Bmal1*: *Per*	3	.78	.46
*Bmal1*: *Ror*	3	1.01	.85
*Bmal1*: *Rev-erbα*	3	.78	.64
*Per*: *Ror*	3	1.29	1.86
*Per*: *Rev-erbα*	3	.99	1.40
**Phase Difference (%Period)**	**Weight**	**Desired**	**Model Feature**
*Per* and PER	10	75	90
*Cry1* and CRY1	5	75	90
*Cry2* and CRY2	5	67	90
*Bmal1* and BMAL1	5	0	7
*Ror* and ROR	5	67	92
*Rev-erbα* and REV-ERB*α*	5	58	82
*Bmal1* and *Per*	15	54	52
*Bmal1* and *Cry1*	15	25	56
*Bmal1* and *Ror*	5	29	57
*Bmal1* and *Rev-erbα*	5	67	79
*Rev-erbα* and PER	50	50	63
*Rev-erbα* and *Ror*	5	79	77
*Per* and *Cry2*	5	0	4
CRY1 and CRY1N	5	0	14
CRY2 and CRY2N	5	0	13
***Cry*** **Knockout**	**Weight**	**Desired**	**Model Feature**
*Cry1* sensitivity	50	<0	-2.46
*Cry2* sensitivity	50	>0	18.0
*Cry1* knockout period	100	<.95	.95
*Cry2* knockout period	100	>1.15	1.16

To ensure the model captured these features of the expression patterns, we fit the 45 parameters of the model using a cost function. This cost function returns a maximum value if the model does not produce limit cycle oscillations, and otherwise the cost function is the sum over all of the features of a difference function of the model produced value and our desired features multiplied by the weight in Table 3. For the numeric values, we use the squared error as the difference function, and for the inequalities, we take the difference function as 0 if the parameter set correctly met the criteria and 1 otherwise. We chose the parameter set which minimizes this cost function. Because the cost function is not convex and we were optimizing a high dimensional parameter set, traditional gradient based algorithms would not be effective. Instead, we used a genetic algorithm, similar to that used in [16]. We initialize a population of 10,000 parameter sets, where initial values for the parameters which correspond to those previously fit in the Hirota model [1] were chosen distributed about the best fit for that model and new parameters are initialized to be a similar order of magnitude to corresponding processes. We implemented this algorithm using the python package DEAP and then allowed it to iterate over 50 generations, where at each iteration, random mutations of individual parameter sets and random crossovers between pairs of parameter sets occur. We found that the algorithm was sensitive to the choice of initial condition as we observed that in many cases the algorithm converged rapidly (in approximately 10 generations) to a local minimum, and so we repeated this process at several different resampled initial points. From these different algorithmic runs, we used the lowest cost parameter set for the model; these parameter values are given in Table 4.

**Table 4:** Model parameters. The parameter set with lowest cost. Because we did not fit the model explicitly to a specific period or to a specific concentration of one of the model species and instead only used relative time differences and relative abundances, the units for both time and species concentration are arbitrary. The amplitude and period can both be arbitrarily scaled to specific experimental results. Units for the rates, denoted with a “v” are arbitrary concentration units per arbitrary time units.

Parameter	Value
*v*_*txn*,*p*_	0.26726
*K*_*txn*,*p*_	0.33468
*K*_*b*_	0.00117
*v*_*deg*,*p*_	0.55011
*K*_*deg*,*p*_	0.00146
*v*_*txn*,*c*1_	0.08212
*v*_*txn*,*c*2_	0.07313
*K*_*txn*,*c*_	0.28353
*v*_*deg*,*c*1_	0.58013
*v*_*deg*,*c*2_	0.52993
*K*_*deg*,*c*_	1.98812
*v*_*txn*,*ror*_	0.08178
*K*_*txn*,*ror*_	0.23693
*v*_*deg*,*ror*_	0.51117
*K*_*deg*,*ror*_	1.20013
*v*_*txn*,*rev*_	1.15003
*K*_*txn*,*rev*_	0.09945
*v*_*deg*,*rev*_	25.99980
*K*_*deg*,*rev*_	2.69984
*K*_*tln*,*p*_	2.00001
*v*_*deg*,*P*_	4.65000
*K*_*deg*,*P*_	0.00031
*v*_*a*,*CP*_	0.00882
*v*_*d*,*CP*_	0.05999
*v*_*deg*,*C*1_	1.59984
*K*_*deg*,*C*_	2.08639
*v*_*deg*,*C*2_	1.51491
*K*_*tln*,*ror*_	0.35918
*v*_*deg*,*ROR*_	1.29973
*K*_*deg*,*ROR*_	1.95961
*K*_*tln*,*rev*_	0.25461
*v*_*deg*,*REV*_	1.29951
*K*_*deg*,*REV*_	1.96010
*v*_*txn*,*ROR*_	1.81951
*v*_*txn*,*REV*_	2.01031
*K*_*txn*,*REV*_	1.72406
*K*_*txn*,*ROR*_	1.07518
*v*_*deg*,*b*_	1.93448
*K*_*deg*,*b*_	0.55369
*K*_*tln*,*b*_	0.37069
*v*_*deg*,*B*_	1.86141
*K*_*deg*,*B*_	2.71612
*v*_*deg*,*C*1*N*_	0.07588
*K*_*deg*,*CP*_	0.05499
*v*_*deg*,*C*2*N*_	0.23010

### Sensitivity analysis

We used sensitivity analysis to understand the features of the model that have the greatest impact on different features of the model’s performance. Of particular interest was how different parameter changes, which could correspond to small molecule inputs, impact the period of the circadian clock and the amplitude of the different species. These effects are captured by the period sensitivity, $\frac{dT}{dp_{i}}$ where *T* is the period of the oscillator and *p*_*i*_ is some model parameter, and the amplitude sensitivity $\frac{dA_{x}}{dp_{i}}$, where *A*_*x*_ is the amplitude of species *x*. We computed each of these sensitivities independently for each parameter locally about the optimal parameter values as determined by the genetic algorithm. The period sensitivity reflects a change in time for the entire oscillation, so is independent of the timepoint at which it is calculated. In contrast, the amplitude sensitivity is dependent on where in the cycle a perturbation occurs, and because we were primarily interested in changes in overall amplitude, unless otherwise stated, we computed the amplitude sensitivity at the peak of the particular species. The methods compute both of these quantities infinitesimally, so that the quantities are independent of choice of range of parameter change and do not need to be normalized by parameter value, are detailed in [17]. Such analysis has been used to understand the robustness properties of different clock architectures [18] and to determine the strongest inputs for control [7]. Based on these results, we were able to make inferences regarding the role of the positive loop of the circadian clock, detailed in the discussion.

### Model predictive control to shift circadian phase

Our model used a system of differential equations to describe the evolution of the expression levels of the different genes and protein species and can be more succinctly expressed by

$$\begin{array}{c} \frac{dx}{dt}=f\left(x\left(t\right),p,u\left(t\right)\right) \end{array}$$ (17)

where $x\left(t\right)\in R^{14}$ are the expression levels of the model states, $p\in R^{45}$, and *u*(*t*) represents a control input to the model, possibly multi-dimensional. We developed our model with the goal of being able to shift the phase of the circadian clock and required that our model have an attractive limit cycle so that we can assign a phase to each point based on the limit cycle, Γ. For a point *x*_0_(*t*) ∈ Γ, we assign a phase *ϕ*(*x*_0_(*t*)) ∈ [0, 2*π*) such that points equidistant in time are also equidistant in phase. Because we required Γ be attractive, we can asymptotically assign phase to any point based on the point to which it converges on the limit cycle, where we define *ϕ*(*x*_*a*_(*t*)) = *ϕ*(*x*_0_(*t*)) for *x*_0_(*t*) ∈ Γ satisfying

$$\begin{array}{c} \lim_{t\to \infty }∥f\left(x_{a}\left(t\right),p,0\right)-f\left(x_{0}\left(t\right),p,0\right)∥=0 \end{array}$$ (18)

One advantage of developing a model to mathematically describe the control of the circadian clock was the ability to predict what inputs could be used to shift the clock and how much these different inputs might shift the clock without having to test the exponentially many combinations of different input combinations, doses, and timing *in vitro*. The most effective combinations could then be tested *in vitro*. Open-loop optimal control policies using light as a single input at the organismal level to shift phase have been developed from model-based predictions of the phase response to light for a simpler model [19]. Because we are interested in developing a multi-input strategy at the molecular level, and because we must contend with the inevitable model uncertainty, we have chosen to apply a multivariable feedback control strategy, model predictive control.

We assumed that the mechanism through which a small molecule input to the clock acts can be represented by changing one or more of the model parameters, allowing us to predict the phase response of the circadian clock to an input by using the infinitesimal parametric phase response curve [20],

$$\begin{array}{c} PRC_{p_{i}}\left(\phi \right)=\frac{\partial^{2}\phi }{\partial t\partial p_{i}}\left(\phi \right) \end{array}$$ (19)

where *ϕ* is the current phase of the model and *p*_*i*_ is the model parameter affected by the input.

Using a first order Taylor expansion, we can then approximate the phase response to an input *u*_*i*_ which acts through *p*_*i*_ by

$$\begin{array}{c} \frac{d\hat{\phi }\left(t\right)}{dt}=\omega +PRC_{p_{i}}\left(\phi \right)u_{i}\left(t\right) \end{array}$$ (20)

where *ω* is the natural frequency of the oscillator, reducing our model to a phase only model to simplify its use in control [21]. We tested that this linear approximation was reasonable in Results. This allows us to use model predictive control (MPC), a closed-loop technique that solves a finite horizon optimal control problem at each stage, to determine an input strategy to shift the phase of the oscillator to align with a reference oscillator *ϕ*_*r*_ through the following algorithm for the multi-input case:

1. **Find a series of control inputs *u* for a time horizon of *N*_*p*_ prediction steps which minimizes**
  $$\begin{array}{c} \sum_{i=1}^{N_{p}}[(w_{i}{\left(\hat{\phi }\left(t\right)-\phi_{r}\left(t\right)\right)}^{2}+\sum_{j}^{J}q_{i,j}u_{i,j}] \end{array}$$ (21)
  **subject to the constraint that *u*_*i*,*j*_ ∈ [0, *u*_*max*,*j*_] where *u*_*i*,*j*_ is the *j*th control input at the *i*th timestep and *w*_*i*_ and *q*_*i*,*j*_ are weights.** To perform this optimization, $\hat{\phi }$ is evolved according to Eq 20, where we perform the optimization using sequential least squares programming in the scipy optimization package, but any optimization algorithm would suffice.
2. **Apply the control inputs *u*_1,*j*_ for all *J* control inputs to the full model.**Rather than using the linearized phase dynamics for $\hat{\phi }$, which simplifies the optimization, we use the full model dynamics to evolve the state of the model to the next time step, using the python package CASADI to perform the integration of the nonlinear differential equations.
3. **Measure the current phase of the model.**We determine the phase of the current state of the model as defined by Eq 18 by integrating the model from the current state forward in time until it reaches within a small Euclidean distance (≤.001) of the limit cycle.
4. **Repeat steps 1–3 for the next timestep**.

Our implementation of this algorithm is also available in the previously cited github repository.

We used this algorithm to compare the trajectories *in silico*. To quantitatively compare the performance of different inputs and the resulting phase trajectories, we used settling time, defined as the earliest time after which the difference between the phase of the model and the phase of the reference trajectory remains within some threshold. (In this work, we choose a threshold of 0.1 radians.) This metric allowed us to compare the speed of different control strategies to align the model with the reference.

## Results

### Model fit and validation

**Fig 2:**
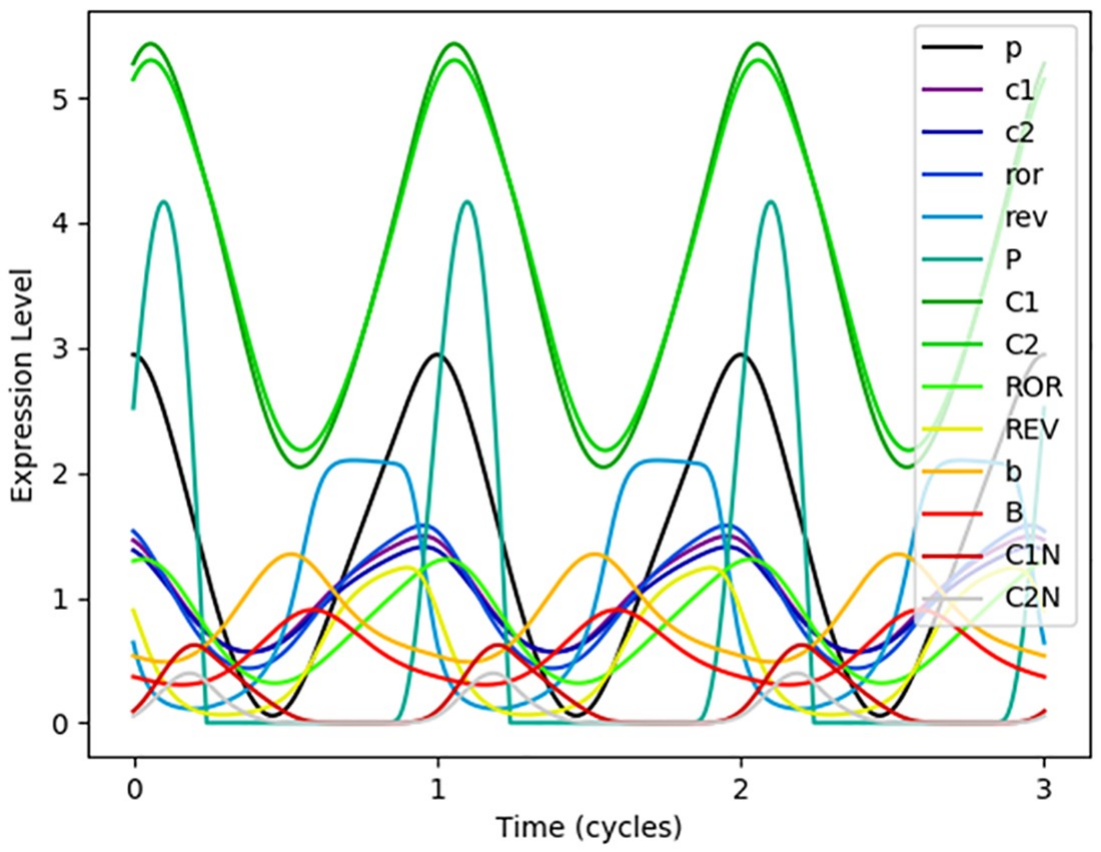
Output oscillations of the model. The output oscillations in expression concentrations of the model parameterized with the lowest cost parameter set show that the model produces a limit cycle. Each model species is represented by a different color line. The x-axis is in arbitrary time units since time can be rescaled to any desired period. From the expression curves, one can visualize the different model features, including peak-trough ratios, relative abundances, and phase differences, used to fit the parameters. Despite similar underlying physiological processes (transcription, translation, and degradation) corresponding to similar equations, each species has a different resulting waveform, presenting a potentially important model outcome for comparison to other models as experimental techniques improve to have better time-resolution for these expression curves.

As outlined in Methods, we found the lowest cost parameter set for the model. The resulting oscillations of the 14 model species are shown in Fig 2. It should be noted that the period of the model is not approximately 24 h as would be expected for a circadian oscillator because we did not explicitly fit the model period. If a certain period is desired, the necessary parameters could be appropriately rescaled in order to rescale time. (The optimal parameter set here without rescaling of time resulted in a period of 27.1 h.) Table 3 shows the features of the expression patterns using this parameter set compared to the desired features as measured experimentally. Since peak to trough ratios, relative abundances, phase differences, and sensitivity to cryptochrome knockout were explicitly included in the cost function, we found that the optimal parameter set gave a good fit to these features. The fit is particularly strong to the peak to trough ratios with relative abundances as areas where the fit could most be improved. Depending on the features of the model which are most important for the performance we consider, such features may be better fit by changing the weights in the cost function.

As a test of robustness of the model fit, we individually varied each parameter by up to 50% to determine the robustness of the fit of the model. We found that 64% of the parameters could be varied in a range of 25% or more without significantly increasing the cost by losing oscillations or the correct responses to cryptochrome knockout, the most heavily weighted features of the cost. Only five parameters (*K*_*txn*,*p*_, *v*_*deg*,*p*_, *v*_*txn*,*c*1_, *K*_*deg*,*c*_, *v*_*deg*,*C*1*N*_), which govern the negative feedback loop, could be varied by less than 1% while keeping the cost low. Using a finite difference approach, we estimated the sensitivity of the cost function to each parameter individually. The parameters to which the cost function is more sensitive could be varied in a smaller range before the cost increased steeply. These dramatic increases are often the result of requiring that the model preserves the correct response to the cryptochrome knockout conditions, so without these conditions, the model fit may be dramatically different. However, within the range of parameters that meet these conditions, the value of the cost function increases by less than 140% suggesting that within a range of different choices of weights for the other model features, the optimal parameter set would still fall near the set we found through the genetic algorithm.

With the exception of the cryptochrome knockout conditions, all of these features were from *in vitro* recordings of non-genetically modified cells. Thus, in order to validate our model, we used *in vitro* observations from experiments which altered the expression of the species in our model. In particular, we test the behavior of our model under knockout conditions by setting the appropriate transcription rate parameters to zero. The results of these validation tests are shown in Table 5.

**Table 5:** Performance on model validation tests. Comparison of the performance of different models on different validation tests. To analyze period (T) changes to increased expression levels of some species, we examine the period sensitivity, expressed as partial derivatives, to the corresponding parameter. Positive sensitivities correspond to an increased period.

Model Validation Test	Dual Loop Model	Leloup and Goldbeter Model	Relogio et al. Model
Model period sensitivity to *Cry* degradation (negative for *Cry1*, positive for *Cry2*)	$\frac{\partial T}{\partial vdc_{1}}<0$ $\frac{\partial T}{\partial vdc_{2}}>0$	Positive for both degradation mechanisms ($\frac{\partial T}{\partial kdmc}>0$, $\frac{\partial T}{\partial dvmc}>0$)	$\frac{\partial T}{\partial y2}>0$
*Cry* knockout period (shorter for *Cry1* KO, longer for *Cry2*)	$\frac{\text{Cry1}\;\text{KO}\;\text{T}}{\text{original}\;\text{T}}<1$ $\frac{\text{Cry2}\;\text{KO}\;\text{T}}{\text{orginal}\;\text{T}}>1$ double KO arrhythmic	arrhythmic	arrhythmic
Increased BMAL1 leads to increased T	$\frac{\partial T}{\partial klB}>0$	$\frac{\partial T}{\partial ksb}>0$	$\frac{\partial T}{\partial kp5}>0$
Increased PER leads to decreased T	$\frac{\partial T}{\partial klp}<0$	$\frac{\partial T}{\partial ksp}<0$	$\frac{\partial T}{\partial kp1}<0$
REV-ERB*α* KO leads to decreased T^#^	$\frac{\text{REV-ERB}\alpha \;\text{KO}\;\text{T}}{\text{original}\;\text{T}}<1$	arrhythmic	arrhythmic
REV-ERB*α* KO leads to decreased *Cry1* amplitude	no change in peak, increased peak to trough ratio	arrhythmic	arrhythmic
*Bmal1* KO causes arrhythmictiy^#^	arrhythmic	arrhythmic	arrhythmic
*Ror* KO leads to decreased *Bmal1*, no *Per* amplitude change	Decrease in peak to trough ratio of *Bmal1* but peak increases, no *Per* change	N/A	arrhythmic
*Bmal1* antiphase with *Per* and Cry*	yes, incorporated in cost function	yes	yes
*Per* KO is arrhythmic*^#^	yes	oscillations possible with increased cooperativity	yes

^#^ Indicates a test used by Relogio et al. [22] for model validation.

*Indicates a test used by Leloup and Goldbeter [23] for model validation.

#### Period sensitivity

Since we were primarily interested in the implications of the model for shifting circadian clock phase, it was particularly important for our model to show the correct period sensitivities to the knockout of different species. In order to ensure that period sensitivities to *Cry1* and *Cry2* knockout were preserved from the Hirota model [1], these features were explicitly incorporated into the cost function with a high weight. As a result, our model continued to display the correct behavior; the knockout of *Cry1* results in a shortened period while the knockout of *Cry2* lengthens the period. We did not explicitly fit the model to other knockout conditions. Experiments show that increased BMAL1 concentrations result in an increased period, while increased PER results in a decreased period [24]. For both of these cases, the sensitivity of the period length relative to the translation rate of these species has the correct sign, so the model correctly reflects this qualitative behavior. Under Rev-erb*α* knockout conditions, the circadian oscillator shows a decreased period (by about 0.5 h in mice or ∼2% [25]), while our model showed only a very slight decrease (<1%).

#### Knockout rhythmicity

Experimental evidence shows that some genetic knockouts render the circadian clock arrhythmic, while in other conditions, oscillations continue, but with changes in period and amplitude. Our model correctly predicted arrhythmicity in *Bmal1* knockout conditions. Under *Rev-erbα* knockout conditions, our model predicted continued oscillations of the negative feedback loop as desired. However, in these conditions *Bmal1* becomes arrhythmic in fibroblast cells, but continues to oscillate in our model [26]. This may result from the fact that the majority of the data used to parameterize our model was based on expression data from liver cells, and recent modeling work on more simplified models suggests that different parameter sets may describe the oscillations of the clock in different organs [27].

#### Amplitude sensitivity

While our model accurately predicts period sensitivity and rhythmicity for most cases, our model was not as successful at predicting amplitude changes under knockout conditions. *Cry1* amplitude should decrease for *Rev-erbα* knockout as well as *Ror* knockout, but there was no change in peak expression with a slight increase in the peak to trough ratio. In the *Ror* knockout case, we also expected reduced *Bmal1* expression, but although the peak to trough ratio decreases, the amplitude of the peak increased. In this case, the model did correctly predict no change in *Per* amplitude [25, 26]. For consideration in control of circadian phase, the correct amplitude sensitivity may not be as important. However, the model may not be as reliable in its predictions for applications in which we are also interested in controlling the amplitude of the circadian clock. For cases in which amplitude sensitivity is important, the model could be refit with these amplitude sensitivities explicitly included in the cost function.

### Model sensitivities

**Fig 3:**
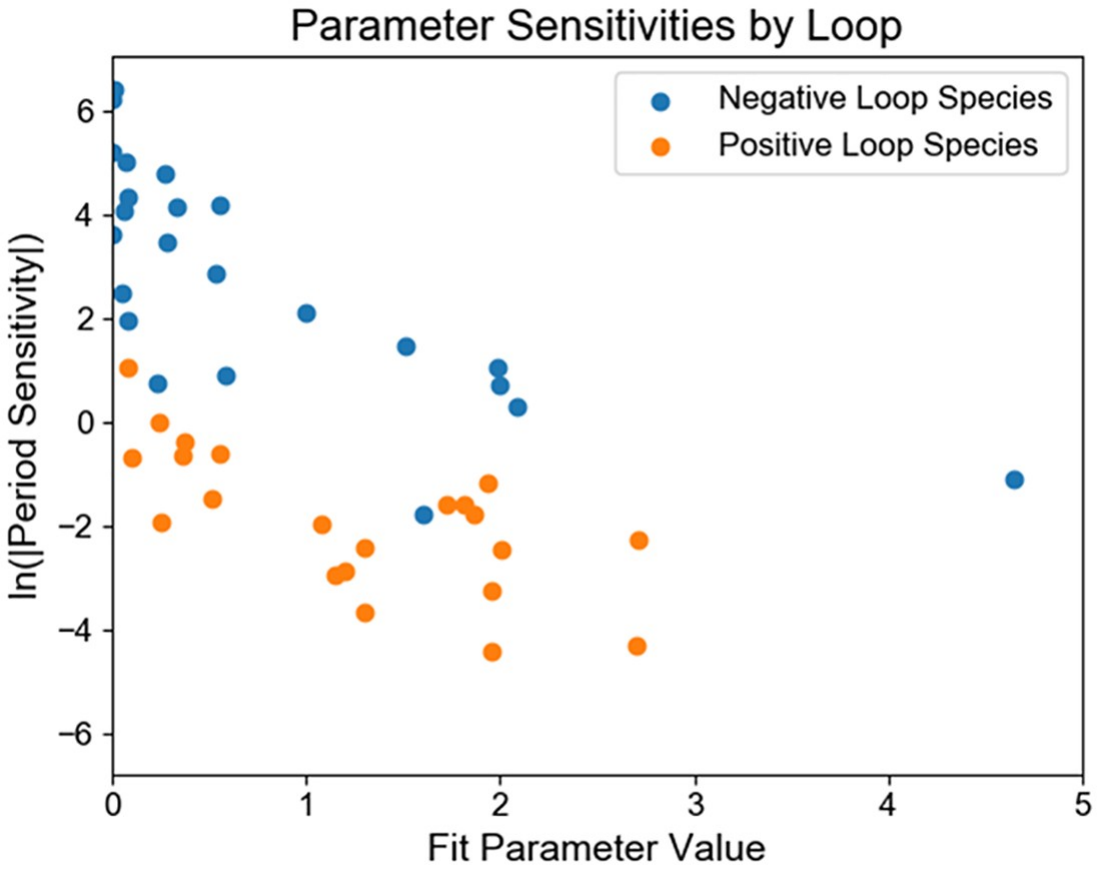
Period sensitivities to model parameters in each loop. For every parameter in the model, we plotted the period sensitivity calculated locally at the optimally fit parameter value versus the fit parameter value. The period sensitivity to the parameters in the negative feedback loop (blue) were orders of magnitude greater than those of the positive feedback loop (orange). Since these sensitivities were infinitesimal, the period sensitivity was largely independent of the magnitude of the fit parameter and depended much more strongly on which feedback loop the parameter governs.

One way to determine which targets may be the most effective for control is to consider period sensitivity to different model parameters. We found that the period of the model is orders of magnitude more sensitive to changes in the parameters of the negative feedback loop than the positive feedback loop (Fig 3). We did not observe any trends of sensitivity to different species within each loop, nor did we find sensitivity differences by function of the parameter, unlike the patterns of sensitivity observed in a model of the circadian clock in Drosophila [28].

**Fig 4:**
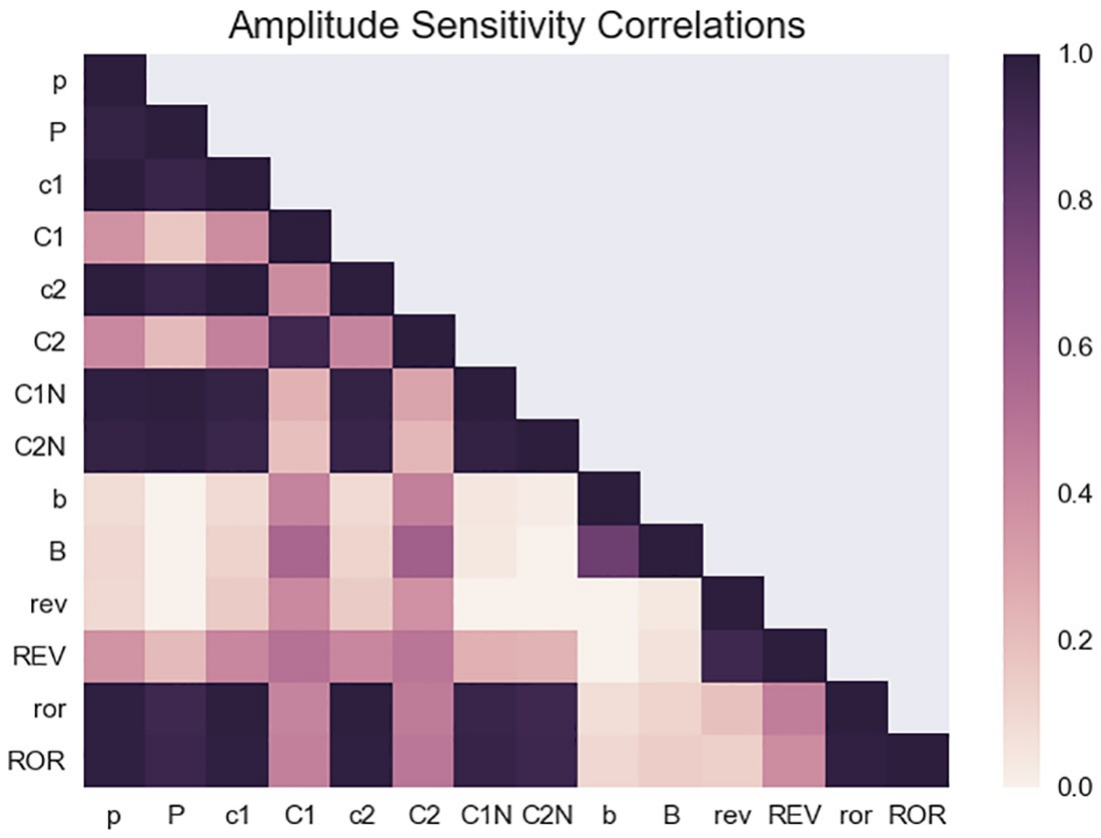
Correlation of amplitude sensitivities of different species to model parameters. For each model species, we computed the amplitude sensitivity for each of the model parameters. The positive correlations between the amplitude sensitivities for each species showed that the amplitude of different species are most sensitive to similar parameters. As expected, these correlations were strongest between the mRNA and protein of the same species. Strong correlations were also seen within the negative feedback loop. These correlations suggested that the targets which are most effective in controlling amplitude for a single species will similarly be strong targets in affecting the amplitude of the other clock species.

Similarly, we observed that amplitude sensitivities to changes in different model parameters were also much more sensitive to changes in the parameters governing the negative feedback loop. The parameters to which the period was the most sensitive corresponded to those which amplitude was most sensitive as has been observed in other models [18]. We analyzed the amplitude sensitivities of each species at the peak expression of that species to each parameter and also found that the similarities in amplitude sensitivity are clustered by species and loop. Unsurprisingly, the amplitude sensitivities of mRNA and proteins were highly correlated as the protein is produced from the mRNA. With the exception of the Ror species, we found a separation of the positive and negative feedback loops again when considering amplitude sensitivity (Fig 4). We similarly found that the cost function was also most sensitive to the parameters governing the negative feedback loop, again demonstrating that most of the dynamic features are driven by the negative feedback loop.

These different sensitivities predict strong differences in the efficacy of different targets for control. We found that the period sensitivities were well correlated with the area under each lobe of the phase response curve (r = −0.92 for the positive area and r = .68 for the negative area). For this reason, we found that the species of the negative feedback loop were able to shift phase much more rapidly with each clock cycle than the positive feedback loop species as described in the next section. More research may consider whether the positive targets are valuable for a more gradual resetting in situations where the light environment cannot be rapidly changed if the shifts from dosing a target of the negative feedback loop are too strong. Moreover, the positive feedback loop targets could be used to make small adjustments to amplitude of the clock as discussed in the next section comparing the phase and amplitude response curves to parameters governing each loop.

### Use of the model for multi-input control

Recent work from our group and others has developed methods for circadian control using a single control input to shift the phase of the clock based on the effects of KL001, which affects the degradation of the PER:CRY1 and PER:CRY2 nuclear heterodimers (corresponding to model parameters *v*_*d*,*C*1*N*_ and *v*_*d*,*C*2*N*_), components of the negative feedback loop [29]. The work of [29] uses the Hirota model [1] so is limited to controlling the clock through small molecules that act on the species in the negative feedback loop. The model developed here allowed us to also use small molecules which act on the species of the positive feedback loop, such as KK-S6 and FBXW7-*α* modulators, which act by reinforcing or degrading REV-ERB*α* respectively (corresponding to changes in *K*_*txn*,*REV*_ or *v*_*d*,*REV*_) [11, 12]. Furthermore, inhibitors of GSK-3*β* act on both arms of the clock, degrading CRY2 and stabilizing REV-ERB*α* (*v*_*d*,*C*2_ and *v*_*d*,*REV*_) [13], requiring a model of both feedback loops of the clock (Table 1).

**Fig 5:**
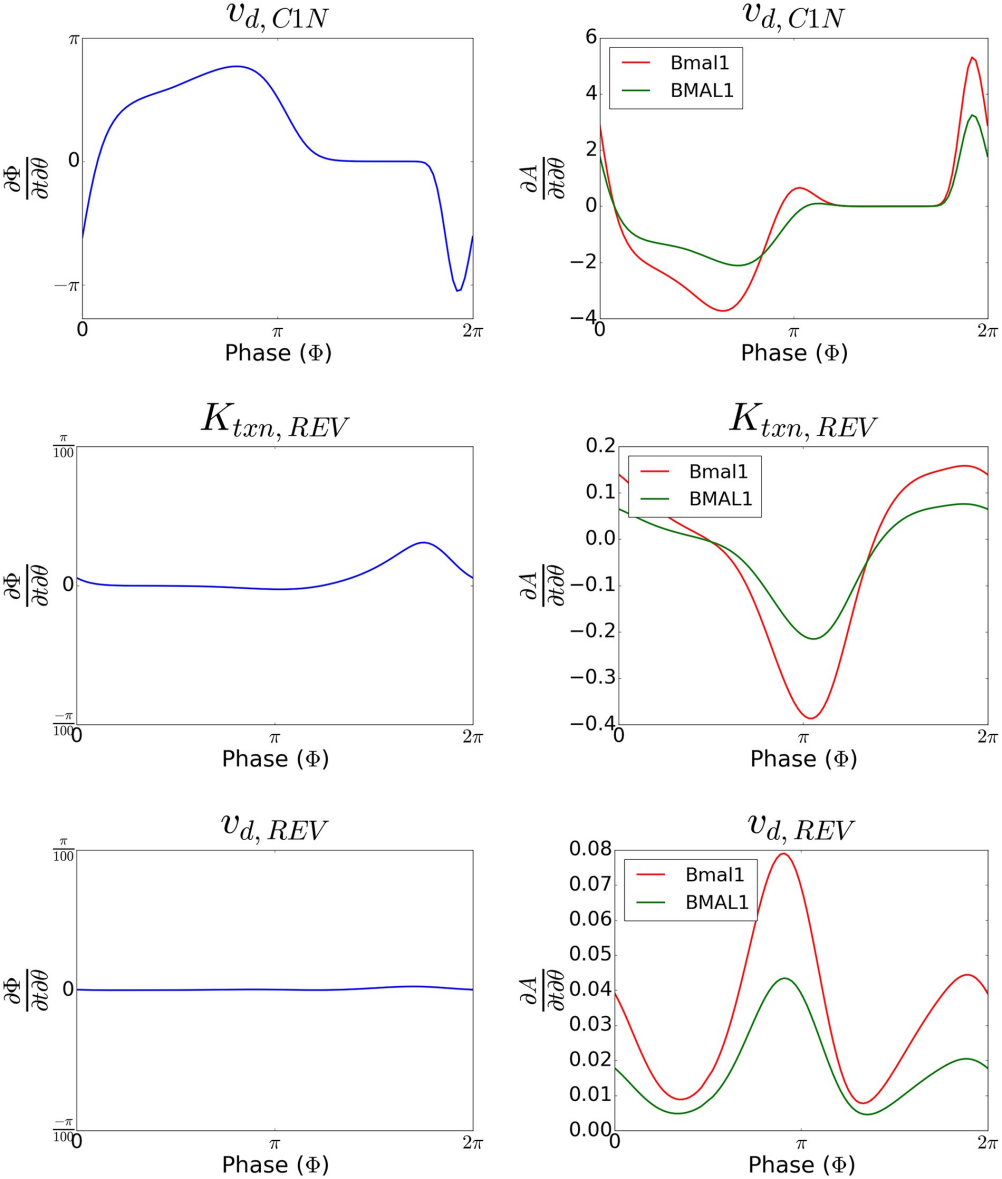
Phase and amplitude response curves to small molecule inputs. The phase response curves (left) and amplitude response curves of Bmal1 and BMAL1 (right) to perturbations in parameters corresponding to the action of known small molecules (Table 1).

As seen in the phase and amplitude response curves of the parameters corresponding to the actions of these small molecules (Fig 5), the magnitude and direction of the phase or amplitude response differs depending on the phase at which the input is applied. This nonlinear response motivated our choice in applying MPC to control circadian phase. From these plots, we also saw the phases at which these different molecules have the greatest impact on the phase or amplitude of the clock differ. This may provide a larger window during which control can be applied to affect the clock. Moreover, these molecules could have complementary effects in controlling for both phase and amplitude; changes to the parameters in the negative feedback loop result in greater magnitude phase responses but also cause changes in amplitude, while changes to the parameters in the positive feedback loop allow for changes in amplitude with minimal impact on phase.

**Fig 6:**
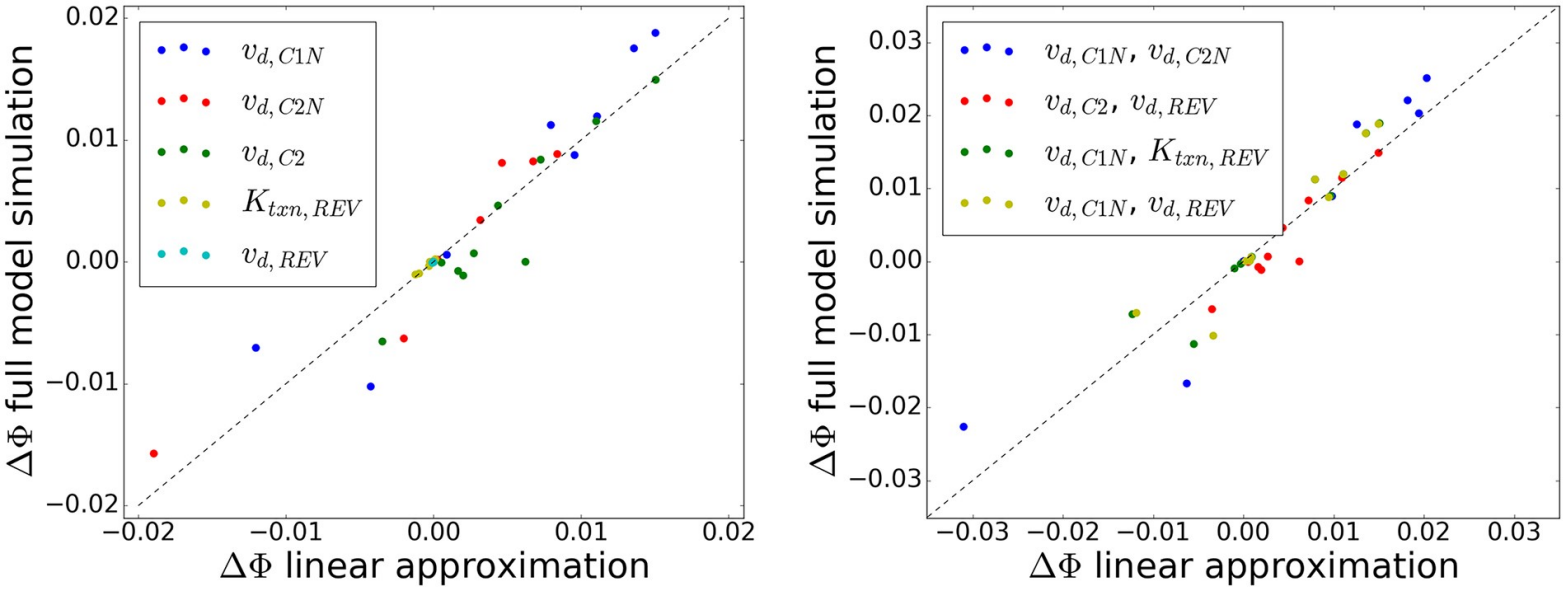
Use of phase only linear approximation to predict phase changes. The phase changes that were observed in the full model compared to the estimated phase changes using the linear approximation from the ipPRC (Eq 20) for a single input (left) or pairs of parameters (right) corresponding to known inputs were strongly correlated, validating the use of this approximation in the MPC algorithm and suggesting that nonlinear interaction terms are not significant for these pairs of inputs.

As outlined in Methods, we used a linear phase-only model to predict changes in phase as the result of control inputs. In Fig 6, we showed that this approximation is valid by comparing the phase change predicted by the phase-only model from the PRC to the phase change of the full model when we manipulated the parameters which correspond to known potential control inputs by 10% for $\frac{1}{24}$ of the period (simulating an hour change). In the single input case, we found a slope of 1.03 with an *r*^2^ of .89, suggesting that this approximation is valid for a single input. Likewise, we found that the linear approximation also held in the case of two inputs, with a slope of 1.05 and *r*^2^ of .89, suggesting that the interaction between the two changes is not significant for phase prediction, allowing us to use this assumption in our MPC algorithm.

**Fig 7:**
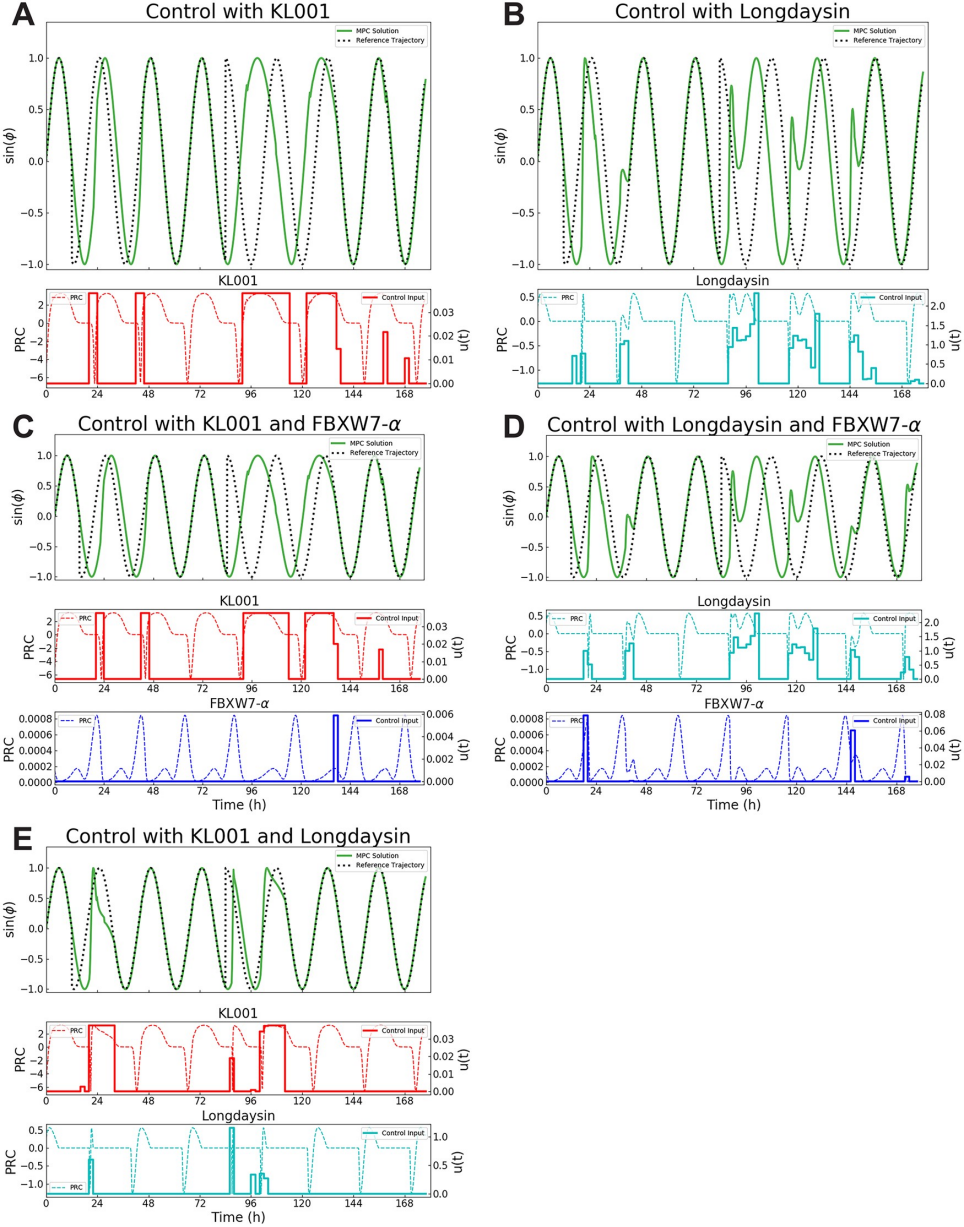
Comparison the MPC algorithm using different control inputs. The output of the MPC simulation using the linear approximation from the ipPRC (Eq 20) for different combinations of parameters. A. KL001 (single input on the negative loop), B. Longdaysin (single input on the negative loop), C. KL001 and FBXW7-*α* (multi-input on the negative and positive loop), D. Longdaysin and FBXW7-*α* (multi-input on the negative and positive loop), E. KL001 and Longdaysin (multi-input on the negative loop). Top panels in each subfigure show the phase trajectory of the model (green) in comparison to the reference (dotted gray). Other panels show the PRC of the control input (dashed lines) and control delivered (solid lines) for KL001 (red), Longdaysin (cyan), and FBXW7-*α* (blue).

We demonstrated the efficacy of the MPC approach with our model to predict phase through simulation of a protocol where a 5 h advance occurs at 12 h into the study and then a 11 h delay is introduced 72 h later using different combinations of inputs (Fig 7). The initial condition of the simulation is set to the peak of *Per* expression. The timing between the phase shifts in this protocol is designed to give the simulation sufficient time to settle in the new phase prior to beginning the delay while phase shifts are chosen to be relatively large so that choices of control input have to be made at many different phases to reach the shifted phase, and the exact choices for the protocol were motivated by our ability to compare with the results from other studies. Previous simulations have used KL001 as control input for the same protocol but using the Hirota model [1], and we found qualitatively similar results to those of [30] when we consider KL001 as a single input to our model. Again for comparison, we use the same MPC specifications with a timestep of 2 h and the control and prediction horizon (*N*_*p*_) both to be 3 timesteps, which were shown to be an optimal tradeoff between computational time and control efficiency for KL001 input to the Hirota model [30]. Slight differences in settling time can be attributed to the fact that the phase response curve for KL001 is not identical for both models, and even for our model, we observed that changes in the model parameterization also cause changes in the phase response curve. To demonstrate the use of the model in multi-input control, we compare the cases of KL001 as a single control input on the negative loop to KL001 with Longdaysin where both inputs act on the negative feedback loop and to KL001 with FBXW7-*α* and Longdaysin with FBXW7-*α* where inputs act on both the negative and positive feedback loop (Table 6). We note that FBXW7-*α* would not have been effective in achieving the desired shifts over this time course due to differences in period sensitivity as discussed in the previous section. The differences in sensitivity were also observed in the fact that control with Longdaysin or KL001 alone was very similar to when we add FBXW7-*α*. While the settling time for the model was similar for KL001 and Longdaysin alone, the trajectory for control with KL001 was much smoother, leaving the questions of what biological relevance such different trajectories have. Finally, the settling time was much faster for the multi-input case using both KL001 and Longdaysin, demonstrating the value in considering questions of multiple control inputs to improve performance. While these simulations demonstrated differences in settling time for different control combinations, we did not optimize the parameterization of the MPC algorithm for each possible input case, instead leaving them fixed for comparison across the cases. Such a comparison showed differences in settling times, depending on the strength of the control input, which could further be refined by optimizing the parameterization of the MPC algorithm for different input combinations.

**Table 6:** Settling time of simulation for different inputs. Comparison of the performance of different input combinations with the MPC algorithm in our simulated scenario on the settling time in the simulation for the 5 h advance and 11 h delay.

Inputs	Target Loop(s)	Advance Settling Time (h)	Delay Settling Time (h)
KL001	negative	45.3	136.2
Longdaysin	negative	42.0	165.8
KL001 and FBXW7-*α*	negative and positive	45.3	136.2
Longdaysin and FBXW7-*α*	negative and positive	42.0	171.1
KL001 and Longdaysin	negative and negative	31.3	101.1

While these *in silico* studies confirmed previous results suggesting that period sensitivity is a good indicator of what inputs are the strongest phase resetting targets [18], these simulations consider phase as the only output. We observed very different trajectories depending on the combination of control input and did not consider controlling for amplitude as a second possible output. Although these results suggest that the parameters governing the positive feedback loop are not as powerful targets for resetting phase, these targets may allow us to control for amplitude nearly independently of phase (Fig 5). Moreover, our simulations assumed that control targets could adjust the parameter by as much as half of its nominal value when control is applied. More experimental data is needed to determine how much a given input can change these parameters and what doses are safe to shift phase without disrupting other biological processes. When considering these constraints, the powerful changes resulting from inputs to the negative feedback loop may not be feasible, resulting in the need to consider more complex combinations of inputs as allowed for by our dual loop model. Our simulations demonstrated the potential for increased speed in achieving phase shifts through multi-input control but with different phase trajectories, which might also be a control target, and our model has been designed to be control-relevant so that it provides the framework to explore these questions.

From the resulting input profiles we also saw, as expected, that the inputs were only delivered when the system was in a state where the phase response curve was either in an advance or delay, the bang-bang control which has been proven to be optimal for this system [30]. This result reaffirmed that not only the dose of an input impacts the system’s response but also the timing of the input, as mistimed inputs can result in shifting phase in the opposite direction than desired. Such results heighten the importance of our ability to control phase so that the delivery of drugs, not only to shift phase but also drugs that act on other related systems, may be timed appropriately for maximal efficacy and motivate the further study into chronotherapy for the effective timing of health interventions.

## Discussion

### Comparison to other models

Numerous previous models have been used to describe the circadian oscillator, ranging from single equation phase only models to large systems of differential equations with over 70 states. Models that are too simple do not have sufficient biological detail to consider different control actions, while models that are too large make determining the most effective control input strategies difficult because of the many combinations of possible targets. For this reason, we sought to develop a model that contained both the positive and negative feedback loop to provide sufficient control targets. This model is most similar in scope to the Leloup and Goldbeter model [23] and the Relogio et al. model [22].

All three of these models contain the core clock species from both the positive and negative loop. Our model has 14 states with 45 parameters, while the Leloup and Goldbeter model has 19 states with 63 parameters, and the Relogio et al. model has 19 states and 71 parameters. Our model has fewer states and parameters because it does not specifically model phosphorylation of the model proteins. As a result, our model loses some potential mechanisms for altering the clock but results in a simpler model that still captures much of the essential behavior. Even with fewer states, our model still had sufficient time delay for oscillations without the additional delay created by adding the phosphorylated states. For cases where the degradation pathway is targeted for phase shifts, our model could be expanded to explicitly include the phosphorylated states to determine an even more precise target process. Aside from the differences in modeling phosphorylation, the models remain similar in size because all of the models assume that similar gene families can be represented as a single state to simplify the number of states. For example all three isoforms of *Period*, *Per1*, *Per2*, and *Per3*, are modeled as a single species representing *Period*. The same is true for *Ror* (not included in the Relogio et al. model) and *Rev-erbα*. A key difference in our model compared to the other two is that our model has separate states for the two cryptochrome isoforms, allowing the model to have different sensitivities to the knockout of one of the genes as is observed experimentally. Instead, the other models both have a positive period sensitivity to the degradation of CRY, as is only the case for CRY1 experimentally. None of the models include CLOCK, and instead assume that there is a sufficient, constant level available for the formation of the CLOCK:BMAL complex. Such an assumption is supported by the fact that CLOCK has been shown to display only minimal oscillations, and moreover, the effects of REV-ERB*α* on CLOCK expression are similar to its effect on BMAL1 [31], so these effects are captured since both REV-ERB*α* and BMAL1 are included in the model.

Unlike our model and the Leloup and Goldbeter model, the Relogio model does not use Michaelis-Menten kinetics to describe the degradation of the model species, making a different mechanistic assumption. Another difference in mechanistic assumption is that our model does not include Hill terms, so does not assume any cooperativity, but still attains sufficient nonlinearity to produce oscillations.

We also considered the performance of these models on the validation tests that we used for our model (Table 5). All three models displayed the correct period sensitivities to PER and BMAL1. Similarly, for all three models, *Bmal1* or *Per* knockout leads to arrhythmicity. One advantage of our model is that it continues to produce oscillations under other knockout conditions as observed experimentally. In addition to the ability to knockout only a single isomer of cryptochrome and have the correct period changes as a result, our model also retains rhythmicity in the knockout of *Rev-erbα* and *Ror* as desired, unlike the other two models. Thus, although the other models provide similar control targets, because the other models do not show the correct period changes in response to all parameter changes, especially in the case of the degradation rates of nuclear cryptochrome where KL001 acts, comparing their performance in our MPC calculation is not well motivated.

Our model also differed in large differences in period sensitivities between the two loops, where the sensitivities to the parameters of the negative feedback loop were orders of magnitude larger than to those of the positive loop as described in Results (Fig 3). This was not observed in either of the other models. In the case of the Leloup and Goldbeter model, this may be partially explained by the fact that it is assumed some of the parameters are common across reactions, necessarily making the two loops more similar to each other. Moreover, the three models do not all predict the same directionality of the period sensitivity to corresponding model parameters, providing other experiments that could be conducted to validate the different models. Previous experiments have validated phase response curves predicted by models describing the oscillators response to light input by delivering pulses of light of different lengths at different phases and measuring the resulting phase change [32]. Analogous experiments could be conducted for the phase response of the oscillator to different small molecules by delivering pulses of the drug to the system and measuring resulting phase changes.

Overall, the dual loop model is advantageous because it is a slightly simpler model while still differentiating the differing effects of the cryptochrome isomers and retaining rhythmicity in cases where it is expected where the other models do not. These properties make our model useful for considering future questions on multi-input control.

### Implications for the role of each feedback loop

Three different functions have been proposed for the positive loop of the clock. First, it was proposed that this additional loop adds robustness to the oscillations. Second, the positive loop may help to coordinate sending environmental signals to the core negative feedback loop. Third, the positive feedback loop may help to coordinate the timing of the expression of specific genes [33]. The results of our modeling and sensitivity analysis provide support for all three potential functions. Our model showed that when the positive feedback loop is incorporated, the Hill terms are no longer needed for the system to have sufficient nonlinearity produce oscillations. This suggests that the positive feedback loop may be necessary to help stabilize oscillations, and that an activator-inhibitor system can produce circadian oscillations as unlike other studies, we do not have a core repressilator loop in our model [14]. Still, the dominance of this repressilator structure with the primacy of the negative feedback from PER and CRY is supported by our findings that the negative feedback loop dominates the positive feedback loop in period and amplitude sensitivities [27]. Taken together, these results also support the findings that additional feedback loops contribute to robustness of the oscillations and to perturbations compared to a single negative feedback loop model [9], suggesting why the biological circadian clock is more complex than is needed to produce oscillations synthetically.

The lower sensitivity of many features of the circadian oscillations to the parameters of the positive feedback loop may also suggest the importance of robustness to these parameters for other functions, largely coordinated by REV-ERB*α* and REV-ERB*β* [34]. The circadian clock is linked to the metabolic system through both *Rev-erbα* and *Rev-erbβ* in a number of different functions [35, 36]. For example, both REV-ERB and ROR have been linked to adipogenesis through their regulation of lipid homeostasis, and ROR is also linked to glucose metabolism, providing the potential to consider known synthetic ligands as therapeutics for metabolic disorders [37]. The positive arm of the clock also links the circadian system to inflammation. In the brain, *Rev-erbα* has been shown to modulate neuroinflammation through microglial activation, and its knockout causes loss of rhythmicity in circadian patterns of neuroinflammation [38]. Similarly, in the rest of the body, *Rev-erbα* regulates the inflammatory response of macrophages by repressing the expression of *Ccl2* [39]. These links between the positive loop and these other systems may suggest that the lower magnitude period sensitivity may help preserve clock function if dysfunction arises in these other systems.

The dominance of the negative feedback loop together with the connection between the positive feedback loop and other systems suggest that when the goal of therapy is to change underlying features of the clock, targets in the negative feedback loop will be more effective. On the other hand, while targets in the positive feedback loop may have smaller impacts on the phase and amplitude of the clock itself, changes in these parameters may have a greater impact on other systems.

### Future directions

Overall, the developed model outperforms existing models in validation, while, importantly for control, maintaining the correct sensitivities to cryptochrome knockout. Since the model contains both feedback loops, the model provides many additional targets for control. Future work should investigate the most effective combinations of multiple inputs as well as a consideration of how they are timed relative to each other. To be most valuable clinically, restricting the inputs to come at the same time would be beneficial.

Moreover, much work remains to be done in the development of useful models for drug timing and drug delivery. Our MPC simulation assumed that an control input could instantaneously cause a parameter change and instantaneously be removed to return the parameter to its original value. Such an assumption ignores the pharmacokinetics and pharmacodynamics of these small molecules, which would require further experimentation to verify, and would change the desired time profile of the inputs. Similarly, the simulation did not account for other naturally occurring environmental inputs to the circadian clock, including light and metabolic cues. Light has been shown to be a valuable control input to shift phase, and as a result, the light environment may change the efficacy of these small molecules [40].

As discussed in the previous section, the clock is linked to many other biological processes. This model also could be incorporated with models of other biological systems to better investigate the relationship between the clock and different body systems. Such linked models could be used to hypothesize whether adverse changes in different body systems result from abnormal expression levels in the clock or whether abnormal physiology can result in dysregulation of the circadian clock.
